# Construction of OH-functionalized MWCNT/solid waste composites with tubular/spherical heterostructures for enhanced electromagnetic wave absorption property†

**DOI:** 10.1039/d2ra01960d

**Published:** 2022-05-27

**Authors:** Mengzhu Liu, Hongwei Wang, Yangyang Lv, Yingyuan Zhang, Yongpeng Wang, Haibo Zhang, Zhenhua Jiang

**Affiliations:** College of Materials Science and Engineering, Jilin Institute of Chemical Technology Jilin 132022 China wyp4889@163.com; College of Chemistry, Jilin University Changchun 130012 China

## Abstract

Electromagnetic wave (EMW) absorption materials with high efficiency and simple preparation process are highly desirable for practical applications. However, there are still many obstacles to simultaneously satisfy the practical requirements. Herein, fly ash cenospheres (FACs), solid waste from power plants, were selected as a framework to prepare OH-functionalized multi-walled carbon nanotube (MWCNT)/FAC hybrids with multilayer, connected and porous architectures *via* a facile physical mixing process for the first time. Accordingly, a novel tubular/spherical model for EMW absorption materials was established. The effect of the unique heterostructure, which possessed multiple interfaces, on the EMW absorption property was studied. The results indicated that this structure is conducive to extending the transmission route, adjusting the conductivity and improving the dielectric loss. Thus, the composite showed an excellent EMW absorption performance. The minimum reflection loss of −44.67 dB occurs at 4.9 GHz and the effective bandwidth below −10 dB (90% attenuation of EMW) could shift from 4.1 to 19.2 GHz with a thickness in the range of 1.5–5.5 mm. The superior absorption property is mostly attributed to the synergistic effect of good impedance matching, multiple loss mechanisms, and multiple reflections and scatterings. Thus, this product meets the requirement of high absorption performance and simple preparation, which greatly enhance its applicability.

## Abstract 2

Novel MWCNT/FAC composites based on a tubular/spherical micro–nano structure system are designed and prepared through physical mixing for achieving enhanced EWA property.

## Graphical abstract

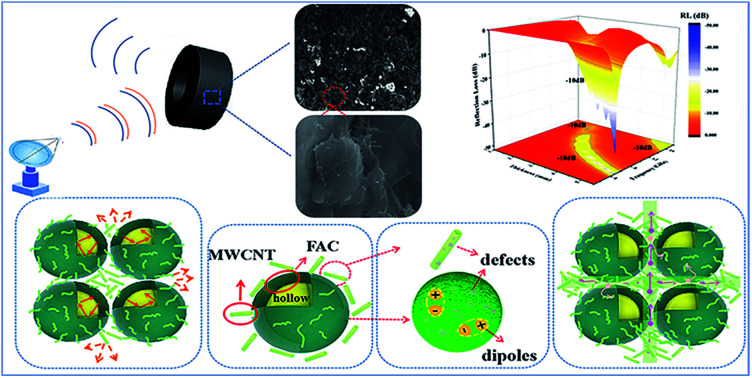

## Introduction

With the rapid development of the modern electronic industry, EMW pollution has seriously increased.^1,2^ Thus, highly efficient EMW absorption materials are urgently required. High efficiency is determined by two factors, *i.e.*, impedance matching and electromagnetic (EM) attenuation capability. Good impedance matching, which can be improved by structural design, ensures that enough EMW enter a material rather than being reflected at its surface. The EM attenuation capability, which is determined by dielectric loss (mainly involving conductive loss and polarization loss) and magnetic loss, guarantees the effective attenuation of the incoming EMW.^3,4^ Therefore, dielectric loss is much more important to enhance the EM attenuation capability for both magnetic and nonmagnetic EMW absorption materials. It has been reported that adequate conductivity is beneficial for EMW attenuation due to the adequate conductive loss, but high conductivity contradicts impedance match.^5^ Excessive conductivity can cause impedance mismatch and lead to a decrease in the EMW absorption performance.^6^ Therefore, designing structural models that can improve the impedance matching and EM attenuation capability by controlling the conductivity and strengthening the dielectric loss is necessary.

Ideal high-efficient EMW absorption materials need to meet the requirements of strong broadband absorption, light weight and thin thickness. For practical applications, the convenient availability and easy fabrication of raw materials are also critical. Many efforts have been devoted to research in this field thus far,^7,8^ but strong absorption is always accompanied by a greater material loading, and high absorption property is always at the expense of complex preparation process. Thus, this severely limits the practical application of absorption materials. Accordingly, to solve the incompatibility problems, the strategy of designing new composites from easily obtained raw materials with simple preparation processes and sufficient dielectric loss is necessary but rarely reported to date.

Firstly, components are critical to guarantee the absorption properties. Carbon-based materials^9–12^ have been chosen as the main raw material to realize good EMW absorption owing to their low density, abundant defects and high specific surface area. Among them, multiwalled carbon nanotubes (MWCNTs) are an excellent choice due to their extra conductivity and special hollow tubular structure.^13^ On one hand, their conductivity can enhance the inner reflections of the incoming EMW to increase the contact between EMW and absorbers, meanwhile providing conductive loss; on the other hand, their hollow structure can further generate multiple reflections to extend the transmission path of EMW, leading to the dissipation of more EM energy. Nevertheless, the single dielectric loss mechanism always limits the absorption property of MWCNTs. Thus, researchers are committed to find ways to solve this problem. Structure design is a key factor to attenuate EM energy.^14^ It has been shown that^2,15–17^ nanostructures with a unique complex morphology are often accompanied by excellent EMW absorption performances due to their extended transmission path for incident EMW, connected network and enhanced interface polarization effect. However, the single tubular morphology of MWCNTs is not sufficient to construct this type of complex structure. Meanwhile, their high conductivity may cause impedance mismatching. Thus, another component with a special skeleton that can adjust their conductivity is necessary. Furthermore, simple processes and low cost are necessary for practical applications. However, it is still a challenge to achieve these capabilities simultaneously. Recently, electrical insulating biomass resources such as bagasse fibres^18^ and bamboo fibres^19^ have been employed as frameworks to fabricate EM interference (EMI) shielding composites by coating them with intrinsically conductive polymers. The insulated natural fibres can act as a bridge to connect the conductive polymers to form a conductive network with superior electrical conductivity. However, this is favourable for EMI shielding, rather than EMW absorption. In the latter case, adequate conductivity is necessary. Thus, if easily obtained materials with a hollow structure, low cost, and high mechanical strength can be used in the structural design of composites with MWCNTs with reasonable conductivity through super simple physical mixing, they will be conducive to enhance the EMW absorption efficiency and improve the applicability of MWCNTs.

Fly ash cenospheres (FACs) are a type of low density, high strength and cheap solid waste from power plants. They are mainly composed of SiO_2_ (56–62%) and Al_2_O_3_ (33–38%), and a small amount of Fe_2_O_3_ (2–4%), SO_3_ (0.1–0.2%), CaO (0.2–0.4%), MgO (0.8–1.2%), K_2_O (0.5–1.1%) and Na_2_O (0.3–0.9%). They have a natural hollow thin wall structure.^20,21^ The amount of –OH groups on their surface can be regarded as the polarization centre of the dipole, which are conducive to enhancing the polarization loss.^17^ Based on the hydrogen bond interaction between the surfaces of FACs, they can be close to each other. Upon dispersing conductive hydroxylated MWCNTs in the voids of FACs, the hydroxyl groups present on the surface of MWCNTs are also readily chemically bonded to FACs. Thus, a thin connected network can be formed. This is different from the core-sheath natural fibre/PANI composites with a thick conductive shell and enhanced pore size.^18^ A large amount of dielectric FACs is encompassed by a thin connected network and the insulating FACs act as a bridge to support the entire conductive network, hindering the movement of electrons due to their insulating property. This will be beneficial to control the conductivity. Therefore, the use of FACs as the framework of MWCNT matrix composites has great advantages. In addition, with the development of the power industry, the waste from power plants is increasing daily, including FACs. The release of numerous FACs in the open air easily causes dust pollution. Furthermore, the burial of waste requires a large amount of land, leading to an increase in environmental pollution and treatment costs.^22^ Therefore, the reasonable use of FACs in EMW absorption materials is not only conducive to improving their EMW absorption efficiency and applicability, but also urgently needed considering the green chemistry concept.

In this work, we constructed a new EMW absorption composite based on a tubular/spherical structure system with high efficiency and strong applicability through the rational utilization of solid waste. A facile effective strategy was designed to construct OH-functionalized MWCNT/FAC composites. Their morphology, microstructural features, EMW absorption performance and fundamental mechanism were systematically investigated. The approach and design promoted herein can provide an idea for the preparation of high-performance microwave absorbents with high practical application value.

## Methods

### Materials

OH-functionalized multi-walled carbon nanotubes (purity > 98 wt%, content of –OH = 5.58 wt%) with an outer diameter of 5–15 nm and length of 0.5–2 μm were purchased from Chengdu Organic Chemicals Co. Ltd, Chinese Academy of Sciences, which were abbreviated as MWCNTs herein. Fly ash cenospheres (FACs) with a diameter of 40–140 μm were provided by Henan Borun Casting Material Co. Ltd Paraffin wax was obtained from Sinopharm Chemical Reagent Co. Ltd.

### Synthesis of OH-functionalized multi-walled carbon nanotube/fly ash cenosphere composites

For the preparation of the OH-functionalized multi-walled carbon nanotube/fly ash cenosphere composites (MFAC), 0.35 g paraffin wax was first weighed and poured into an agate mortar, and then 0.05 g MWCNTs was added. After they were fully mixed through grinding, 0.60 g FACs was added to the same agate mortar. Then, the sample was ground a second time. Subsequently, the composites were pressed into a regular cylindrical shape with a thickness of ∼2.0 mm. A black composite bulk with 65 wt% loading of sample was obtained. All the above-mentioned operations were very easy and did not require any other chemical treatment. Paraffin wax was applied as the substrate because it is transparent to EMW and has no effect on EMW absorption.^23^

For comparison, pure MWCNTs and FACs were also mixed with paraffin wax to study their EMW absorption properties, which were abbreviated as MWCNTs and FACs, respectively. In the MWCNT samples, the ratio of MWCNTs and paraffin was 5 : 95, whereas that in the FAC sample was 60 : 40. The amounts of raw materials were the same as that used for MFACs.

### Characterization

Field-emission SEM (SU-8020, Hitachi, Japan) was employed to characterize the morphology of the samples. The nitrogen adsorption–desorption isotherm and BJH analyses were performed on a Micromeritics ASAP 2460 analyser. The X-ray powder diffraction (XRD) pattern of the prepared composites were measured on a Siemens X-ray diffractometer (D5005XRD) with Cu Kα radiation (*γ* = 1.5418 Å) operating at 40 kV and 30 mA to analyse their crystal structure in the scan range of 10–60° (2*θ*). Raman spectroscopy (Horiba LabRAM XploRA, France) with an He–Ne laser (532 nm) was used to measure and analyse the Raman spectra of the samples. A Fourier transform-infrared (FT-IR) spectrometer (SHIMDZU, 1.50U1, Japan) was employed to identify the vibration of the functional groups present in the samples. Electrical conductivity was measured using an Agilent Precision Impedance Analyzer (HP4294A) equipped with a variable temperature oven (EC1A). The sample was first placed in the middle of a double-layer aluminium plate with an aperture of 10 mm, and then steamed in a high vacuum evaporator (ZHD300). The thickness of the metal copper electrode was about 50 nm and the diameter of the round copper electrode was 10 mm. The electromagnetic properties of the specimens were analysed using a network analyser (VNA, Keysight Technologies, E5071C, USA) through the coaxial method at a band of 2–20 GHz. The samples were cut into a ring shape with an outer diameter of 7.0 mm and an inner diameter of 3.04 mm using a toroidal-shaped mold. Reflection loss (RL) was computed using the transmission line theory.

## Results

### Morphology

**Fig. 1:**
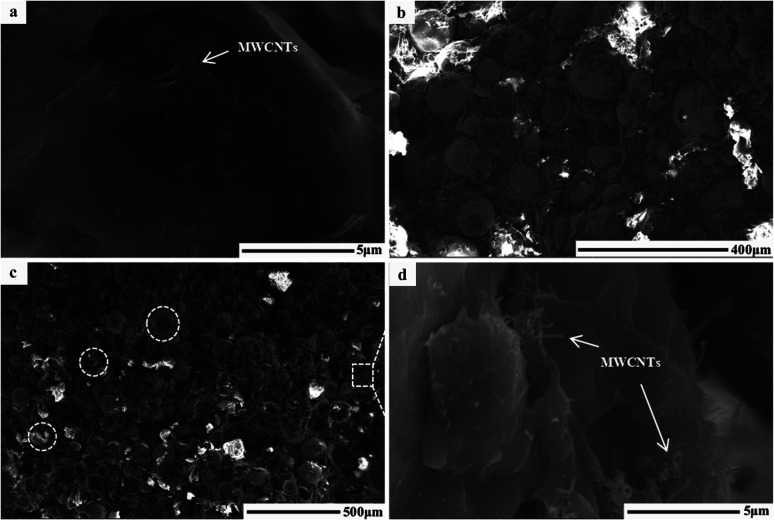
SEM images of the ring used to characterize the EMW absorption properties. (a) MWCNTs, (b) FACs, and (c) MFACs, and (d) is partial magnification of (c).

**Fig. 2:**
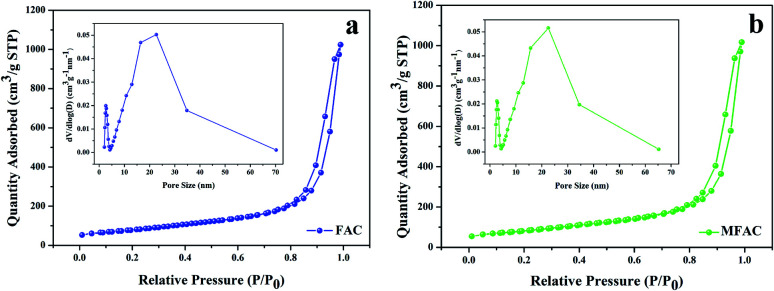
N_2_ absorption–desorption isotherms with pore size distribution curves for (a) FACs and (b) MFACs.

Fig. 1 shows the morphology of the samples. In the ring of MWCNTs with paraffin (Fig. 1a), it can be seen that MWCNTs with an average diameter of 81 nm are dispersed uniformly. After grinding, the MWCNTs maintained their nanotube structure. Their interfaces are mainly composed of the inner nanotube surface and outer nanotube surface. In the case of FACs (Fig. 1b), a few spheres were broken by grinding but most retained their original shape. The spheres were hollow and dispersed in the paraffin homogeneously. There were also two interfaces, the inner sphere surface and outer sphere surface. Their surface was rough with paraffin and the gaps between the spheres formed numerous pores. The mean diameter of the spheres was as large as 62.6 μm. Due to their huge size difference, after physical mixing of MWCNTs and FACs, the morphology of the composite was similar to that of pure FACs (Fig. 1c). The mixture also retained a good morphology after grinding, indicating that the composite had good mechanical properties. The spheres were arranged by the interaction of their functional groups, and then fixed and connected by paraffin. In the region circled by a white dotted line, it is obvious that the spheres were hollow. In the gaps (Fig. 1d), it can be seen that MWCNTs mainly distributed on the surface of FACs and some of the nanotubes tangled together because of the hydrogen bonds between the hydroxyl functional groups of the two components. Due to the contact with MWCNTs, a thin connected network was formed. This is beneficial for the conduction of electrons. In the case of the unbroken FACs, all the MWCNTs were located outside the cenospheres. This is because the composite was prepared by physical mixing and the MWCNTs could not enter in the cenospheres. In contrast, for the broken FACs, some MWCNTs were located on their inside (Fig. S1†) due to the interaction of their functional groups. This is beneficial to provide more chances for electromagnetic waves to contact the materials. It was also detected that the composites maintained a porous structure, with pores distributed inside and outside FACs. The outer pores were generated from the contact between the spheres, which was the same for MWCNTs. The inner hollow tubular structure and outer pore distribution determined the porosity. The porosity was characterized by N_2_ absorption–desorption isotherms. Due to the given specific surface area of the MWCNT raw material, which was >380 m^2^ g^−1^, only FACs and MFACs were characterized. It is obvious from Fig. 2 that the isotherms of both of FACs and MFACs were type IV, indicating their mesoporous characteristics and presence of relatively large pores in these specimens.^24^ The BET surface area and pore volume of FACs were 286.5575 m^2^ g^−1^ and 0.912 822 cm^3^ g^−1^, which for MFACs increased to 288.2802 m^2^ g^−1^ and 1.307 876 m^3^ g^−1^, respectively, indicating an increase in the specific surface area after the formation of the composite. This was also beneficial to enlarge the electromagnetic wave path.

Simultaneously, a new interface was generated between the spheres and nanotubes. The various structures can result in the formation of many heterogeneous interfaces and defects, which are beneficial for interface polarization and dipole polarization. Furthermore, the holes and the pores can act as dihedral angles to enhance microwave scattering and reflection.^25^ By combining a material with a hollow structure, the density of the material can be reduced and its specific area can be increased. It has been reported that samples with a low density can possess a relatively high density of micro-conductive networks according to the conductive network model and the mechanism of aggregation-induced charge transport,^26^ thereby effectively enhancing the conductive loss. Therefore, it can be deduced that the composite in which MWCNTs partly aggregated had enhanced conductive loss.

### Structure

**Fig. 3:**
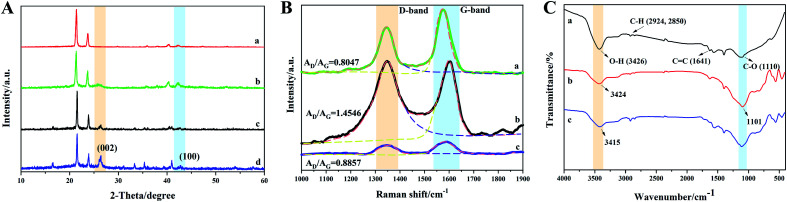
(A) XRD patterns of the ring used to characterize the EMW absorption properties: (a) pure paraffin wax, (b) MWCNTs, (c) FACs, and (d) MFACs. (B) Raman spectra and (C) FT-IR spectra of samples without paraffin wax: (a) MWCNTs, (b) FACs, and (c) MFACs.

Given that the final samples were compounds of the as-prepared samples with paraffin wax, their XRD patterns were directly tested, and in Fig. 3A, where pure paraffin wax was characterized for comparison. Then, the peaks corresponding to the pure paraffin wax were ignored in the other three samples. Thus, in Fig. 3A(b–d), the typical reflection peak at 25.8° can be seen. This reflection corresponds to the (002) planes of crystalline graphite-like materials.^27^ The other typical peak of graphite structures at the 2*θ* angle of 43° is attributed to the C (100) plane.^28^ Thus, these results indicated that grinding did not destroy to the crystal structure of MWCNTs. Besides the peaks of paraffin, the residual peaks in the MFAC compound were mainly caused by the diffraction of FACs, which were composed of SiO_2_, Al_2_O_3_, Fe_2_O_3_, CaO, MgO, K_2_O, Na_2_O, TiO_2_, SO_3_, P_2_O_5_, *etc.*^29^

Fig. 3B shows the Raman spectra of all the samples. For carbon materials, the two peaks in their Raman spectra, which are called the D band and G band, can be seen at 1340 cm^−1^ and 1590 cm^−1^, respectively. Due to the big influence of paraffin wax on the samples, the peaks belonging to carbon materials could not be recognized clearly (shown in Fig. S2†). Thus, the pure components without paraffin wax were characterized. In general, the D band is caused by defects and disorder. Especially, the width of the D band represents the degree of disorder. It was obvious that after the combination of MWCNTs and FACs, the D band became wider, which indicates that the crystal structure of the material exhibited great disorder and there were many defects. The G band is caused by sp^2^ hybridised carbon. It represents the order of graphite, which provides some evidence for the presence of graphite. The ratio of *A*_D_/*A*_G_ represents the degree of graphitization of materials.^30,31^ The values of *A*_D_/*A*_G_ for MWCNTs, FACs and MFACs were calculated to be 0.8047, 1.4546 and 0.8857, respectively. The *A*_D_/*A*_G_ value of MFACs was between that of MWCNTs and FACs. This may be related to the number of defects. In the case of MWCNTs, due to their crystalline structure, they had less defects than the amorphous structure. In the case of FACs, many vacancy defects were left during the process of high temperature carbonization, which removed a large number of oxygen atoms. The residual oxygen atoms existed in the form of asymmetric oxygen-containing groups, which disturbed the sp^2^ state of graphite, leading to amorphous and low graphitization of the samples.^32^ The physical mixing caused the MFACs to have a moderate value, which was slightly higher than that of MWCNTs. This is attributed to the fact that the multiple interface structure and addition of FACs can result in the formation of more defects. These defects can act as the polarization centre of the dipole, which can produce dipole-turning polarization under the action of an EM field.^33^ This is beneficial for enhancing the polarization loss. Moreover, the defects in the graphite layer may produce additional states near the Fermi level, which would cause the absorption of microwaves through the adjacent states on the Fermi level. Thus, it can be speculated that the obtained composite nanofibers may have great polarization loss ability.

To provide insights into the interaction between MWCNTs and FACs, an FT-IR investigation was carried out. To eliminate the influence of paraffin wax, the pure components without paraffin wax were characterized, and the results are shown in Fig. 3C. For pure MWCNTs, the appearance of a wide band at 3426 cm^−1^ is attributed to the hydroxyl groups (–OH). The peaks at 2924 cm^−1^, 2850 cm^−1^, 1641 cm^−1^ and 1110 cm^−1^ correspond to the vibrations of C–H, C=C, and C–O, respectively.^34,35^ The results demonstrate that there were many hydroxyl groups on MWCNTs, which is consistent with their original structure. For pure FACs, two distinct peaks at 3424 cm^−1^ and 1101 cm^−1^ belonging to the vibration of –OH and C–O can also be observed, respectively. After mixing, the characteristic peaks of both MWCNTs and FACs could be found, demonstrating their successful combination. However, the peaks associated with the –OH groups and C–O shifted to 3415 cm^−1^ and 1111 cm^−1^, respectively. The slight shift in these peaks indicates the interaction between MWCNTs and FACs. The free –OH on both MWCNTs and FACs rapidly formed intermolecular hydrogen bonds,^36^ which facilitated the formation of a connected network.

### Conductivity

**Fig. 4:**
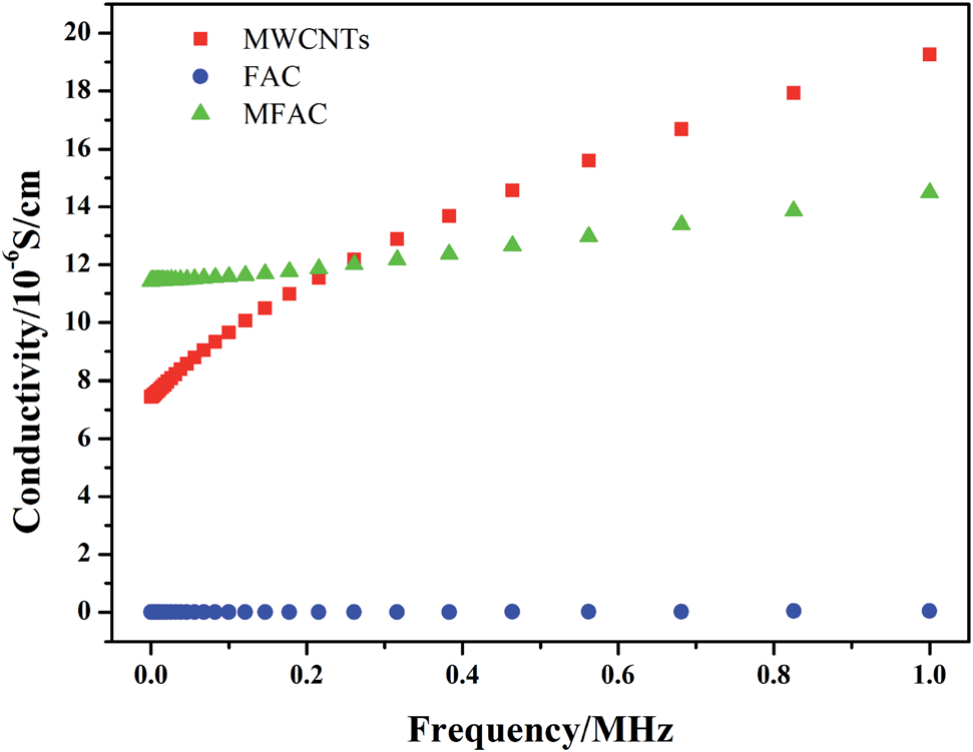
Dependence of the conductivity of the different samples on frequency, measured at room temperature.

Electrical conductivity is one of the critical factors to verify the formation of connected networks. Thus, the change in the conductivity of the three samples with frequency was detected, as shown in Fig. 4. It can be seen that pure FACs were not conductive, while the obtained composite exhibited the highest conductivity initially. In the system considered in this study, the conductivity was only dependent on the MWCNT layer because FACs are an insulating material. As previously discussed, a continuous layer of MWCNTs was constructed on the surface of FACs (Fig. 1d). Thus, the conductivity result is consistent with the structural observations. The connected MWCNT layer contributed to the integrity of the conductive network. On one hand, the FACs in the composite acted as a bridge to connect the nearby MWCNTs and support the entire conductive network; on the other hand, induced the formation of pores.^18^ Thus, an effective charge transport route was established and a conductive system was constructed. However, the content of MWCNTs was so small that only a thin conductive network could be obtained. Compared with the pure MWCNTs, although they had a connected network that was beneficial for the transport of electrons, the composite contained a large amount of insulated FACs. Thus, the conductivity decreased in the following process. This was beneficial to obtain adequate conductivity to ensure good impedance matching.

### Electromagnetic parameters

**Fig. 5:**
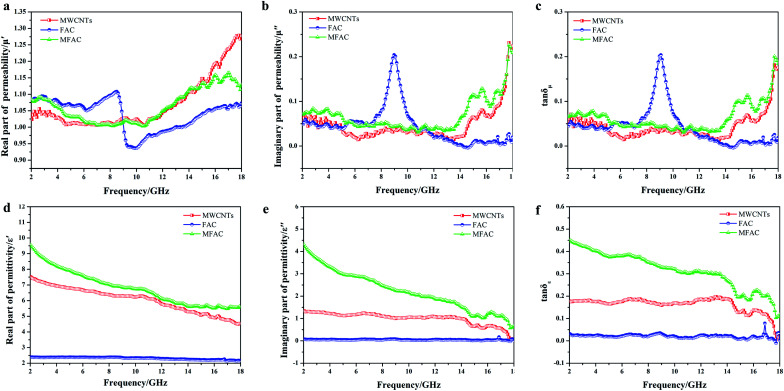
(a) Real parts (*μ*′) and (b) imaginary parts (*μ*′′) of complex permeability of MWCNTs, FACs and MFACs. (d) Real parts (*ε*′) and (e) imaginary parts (*ε*′′) of complex permittivity of corresponding samples. (c) Magnetic loss tangent and (f) dielectric loss tangent of corresponding samples *versus* frequency.

Considering the close association of EMW absorption properties and permittivity and permeability, the frequency dependence of the real part, imaginary part and loss tangent of MWCNTs, FACs and MFACs in the frequency range of 2–18 GHz was studied. Fig. 5a and b describe the real part (*μ*′) and the imaginary part (*μ*′′) of the complex permeability of the three samples. The curves of *μ*′ and *μ*′′ *versus* frequency exhibited relatively strong fluctuation in the frequency range of 2–18 GHz. MWCNTs and MFACs exhibited some similar peaks. This may be attributed to the eddy current effect at the nanoscale^37^ and the magnetic moment in disordered carbon materials.^38,39^ FACs showed some obvious fluctuations before ∼6 GHz and the most intensive fluctuation in the range of 8–10 GHz. It has been reported that exchange resonance occurs at a higher resonance frequency than natural resonance according to Aharoni's theory.^40^ Moreover, MFACs showed similar peaks with FACs before ∼6 GHz. Thus, it may be reasonable to deduce that the former resonance peaks were due to natural resonance, while the latter corresponding to a higher frequency is due to exchange resonance.^41^ However, after the combination of MWCNTs and FACs, the peak corresponding to exchange resonance shifted to 16.8 GHz and its intensity decreased. The significant increment in the frequency may be due to the small size of the MWCNTs in the composites.

According to Aharoni's theory, the exchange resonance frequencies are given by *f*^ exch^_res_ = *f*^ nat^_res_ + *γCu*^2^_kn_/*R*^2^*M*_s_, where *f*^ nat^_res_ is the natural resonance frequency of magnetic particles, *γ* is the gyromagnetic ratio, *C* is the exchange constant, *u*_kn_ is the eigenvalue of the derivative of the spherical Bessel function *j*_n_(*u*), *R* is the radius of the magnetic particles, and *M*_s_ is the saturation magnetization. For isotropic magnetic materials, *f*^ nat^_res_ can be expressed as *f*^ nat^_res_ = *γM*_s_/[3π(*μ*_i_ − 1)],^42^ where *γM*_s_ is the Snoek constant, and *μ*_i_ is the initial permeability. Thus, *f*^ exch^_res_ = *γM*_s_/[3π(*μ*_i_ − 1)] + *γCu*^2^_kn_/*R*^2^*M*_s_. It can be seen that it is difficult to simultaneously increase the magnetic permeability and resonance frequency. Therefore, the intensity of the peak that shifted to a higher frequency decreased. The magnetic loss tangent can directly reflect the level of magnetic loss. It can be seen that the values of all of the samples were close to 0 (Fig. 5c), implying that they had negligible magnetic loss. This is consistent with the component of the samples that was non-magnetic. For nonmagnetic dielectric absorbers, the *ε*′ and *ε*′′ values must be considered.

As observed in Fig. 5d, the *ε*′ of all the samples showed a dielectric dispersion, which can be attributed to the hysteresis of polarization caused by the change in electric field.^17^ For FACs, both *ε*′ and *ε*′′ were very low (*ε*′ = 2.37–2.14 and *ε*′′ = 0.09–0.04) (Fig. 5d and e, respectively) with nearly no fluctuation, demonstrating their poor dielectric loss property. The slight variation in MWCNTs and MFACs can be ascribed to their chemical structure and microstructure.^43^ Comparing the two pure raw materials, the *ε*′ of MFACs was significantly higher. This distinct change may be directly related with the change in its microstructure (Fig. 1), which produced some synergistic effects. For FACs, there were two interfaces, sphere inner surface and sphere outer surface, which were the same for MWCNTs. After the two materials mixed, the interfaces became more. In the hollow spherical nanotube structure, besides the original inner and outer surface of the sphere and nanotube, there was an additional contacted interface. The hanging bond on the interface of the hollow spherical nanotube structure can bind more space charge, thus leading to an increase in the storage of electrical energy and *ε*′.^44^ It has been reported that in the microwave band, the polarization loss mainly included interface polarization and dipole polarization.^17^ Therefore, interface polarization was produced by the aggregation of space charge at the heterogeneous interfaces, which then dissipated the electrical energy. The abundant polar functional groups in FACs (such as hydroxyl group) can be regarded as the polarization centre of the dipole to generate dipole polarization, and then consume the EMW. MFACs possessed the most interfaces, which were beneficial to form multi polarization and enhance the space charge conduction. Thus, their *ε*′′ was much larger than that of the other two materials. The dielectric loss tangent values of the different samples are shown in Fig. 5f. MFACs exhibited the most obvious change with frequency (from 0.45 at 2 GHz to 0.11 at 18 GHz), which represents its strong dielectric loss capacity and excellent high frequency response. On the one hand, the multiple interfaces produced from their complex structure can increase the storage and loss of EM energy, which is much higher than that of a single structure. On the other hand, the large specific surface and high conductivity of MWCNTs in the composite helped to construct a conductive network to promote loss. Therefore, the incident EMW was further attenuated. In addition, it can be found that all the tan *δ*_ε_ values were greater than tan *δ*_μ_ in the tested range, which indicates that the dielectric loss is the dominant factor in the absorption mechanism.

## Discussion

### The origin of the EMW absorption properties

To determine the origin of the EMW absorption properties of the samples, the relaxation process related to the Cole–Cole semicircles was studied. Based on the Debye relaxation theory, *ε*′ and *ε*′′ can be determined according to eqn (1), as follows:1
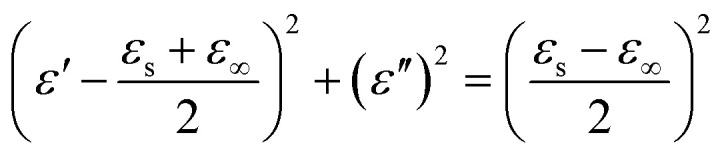


**Fig. 6:**
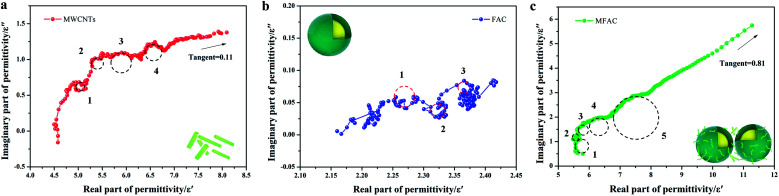
Cole–Cole semicircles of (a) MWCNTs, (b) FACs and (c) MFACs.

The Cole–Cole semicircle was derived from the plot of *ε*′ *versus ε*′′ according to eqn (1). Each semicircle represented the Debye relaxation process, which is related to interfacial polarization, electron relaxation polarization and dipole relaxation polarization. As observed in Fig. 6, all three samples exhibited many relaxation processes, implying that the polarization relaxation mechanism had played an important role in microwave energy attenuation. For the pure carbon nanotubes, they contained defects and polar groups, which provided conditions for dipole polarization.^17^ Moreover, the hollow structure of MWCNTs caused them to have enough room for the accumulation of space charges, similar to FACs. Thus, both of the pure raw materials had some relaxation processes. By comparison, the composite exhibited five relaxation processes, which were the most among the three samples. It has been reported^45^ that the electronic relaxation frequency is generally higher than the dipole relaxation frequency due to the shorter relaxation time of electrons. As the permittivity decreases with an increase in the relaxation frequency, the small semicircle corresponding to low permittivity represents electronic relaxation polarization, while the larger semicircle represents dipole relaxation polarization. It was also easy to detect that the semicircles widths of the composite were larger than that of the pure raw materials. This demonstrates that the combination improved the intensity of the Debye dipolar relaxation process.^46^ It is known that space charges can more easily accumulate at the interfaces between different media. Thus, the additional bigger circle was mainly due to the increase in interface polarization.^47^ In the case of MFACs, the interface polarization relaxation would be produced at the carbon nanotube inner and outer layers, FAC inner and outer layers and the interface of the carbon nanotubes and FACs. In addition, a linear tail can be seen at the end of the Cole–Cole semicircle curve for MWCNTs and the composite. The slope of the linear part always represents the degree of the conductive loss of the materials, which is a factor that influences their EMW absorption properties. According to Fig. 6, the tangent of the linear part for the carbon nanotubes and the composite was 0.11 and 0.81, respectively, indicating that the composite possessed greater conductive loss. Thus, these results support our deduction in the above-mentioned morphology analysis. Although the large amount of insulative FACs caused the overall conductivity to decrease, the partial aggregation of the conductive MWCNTs, which formed a connected conductive network, was beneficial to enhance the inner conductive loss.

### The EMW absorption properties

To evaluate the EMW absorption properties, the reflection loss (RL) of the samples was calculated using the measured complex relative permittivity and permeability according to eqn (2)–(6) based on the transmission line theory and metal back-panel model, as follows:2
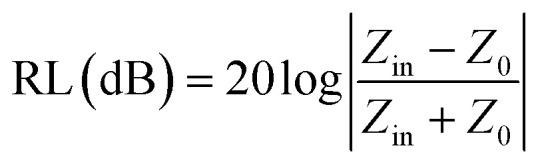

3 *Z*_in_ = *Z*_0_*Z*_r_ tan *η*[ *j*(2π*fd */*c*)(*μ*_r_*ε*_r_)^1/2^]

4 *Z*_r_ = *Z*/*Z*_0_ = 1/(*ε*_r_/*μ*_r_)^1/2^

5 *ε*_r_ = *ε*′ − *jε*′′

6 *μ*_r_ = *μ*′ − *jμ*′′

where *Z*_in_, *Z*_0_, *Z*_r_, *Z*, *f*, *d* and *c* represent the input impedance of the absorbent in the atmosphere, impedance of the free space, impedance matching ratio of the absorbent, impedance value of the absorbent, frequency of the electromagnetic wave, coating thickness, and velocity of the EMW in free space, respectively.

**Fig. 7:**
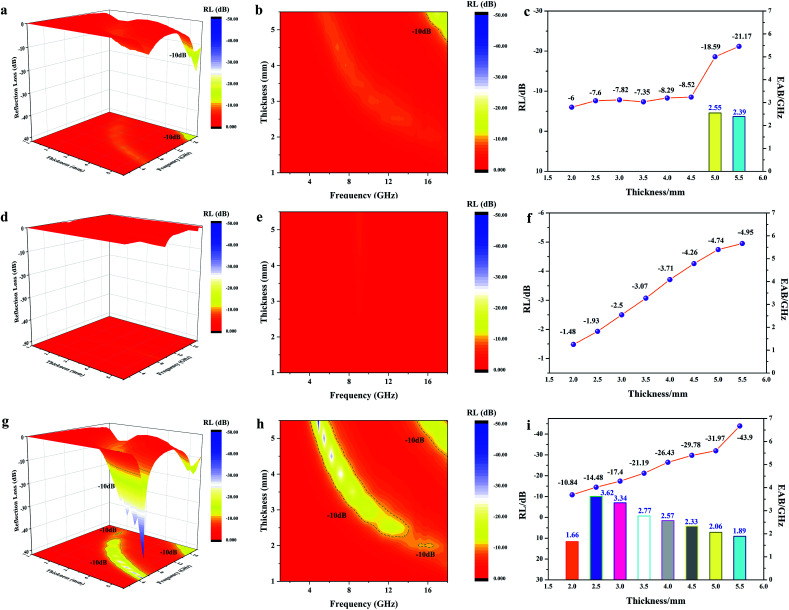
3D RL map, 2D RL contour map, RL value and EAB value in the thickness range of 1.5 mm to 5.5 mm for MWCNTs (a–c), FACs (d–f) and MFACs (g–i).

Generally, an RL value of less than −10 dB can be considered as effective absorption, in which over 90% of the EMW was lost. The corresponding bandwidth is called the effective absorption bandwidth (EAB). The three-dimensional and two-dimensional reflection loss maps of all the samples and the RL and EAB values under the corresponding thickness are shown in Fig. 7, where the effective absorption part is plotted as a line. For pure MWCNTs, it can be seen that when their thickness was below 4.5 mm, there was no effective absorption. With an increase in the sample thickness, the effective RL values gradually increased with frequency. The minimum value was −21.17 dB at 5.5 mm, while the EAB increased initially, and then decreased, reaching the widest 2.55 GHz at 5 mm (Fig. 7a–c). The absorption of FACs was worse. In the entire frequency range, there was no effective absorption (Fig. 7d–f). The best RL value was only −4.95 dB at 5.5 mm, demonstrating that pure FACs are unsuitable for EMW absorption. However, it was surprising that MFACs exhibited a remarkable EMW absorption property. The minimum value reached −44.67 dB at 5.5 mm and their EAB reached the widest 3.62 GHz (10.68–14.30 GHz) at 2.5 mm, whose trend was increasing initially, and then decreasing (Fig. 7g–i). It is obvious that the combination of carbon nanotubes and the hollow FACs resulted in a qualitative leap in EMW absorption property. This is attributed to the multiple reflections and scatterings caused by their special morphology and the synergistic effect of the polarization loss and conductive loss, as discussed in Fig. 5 and 6.

**Fig. 8:**
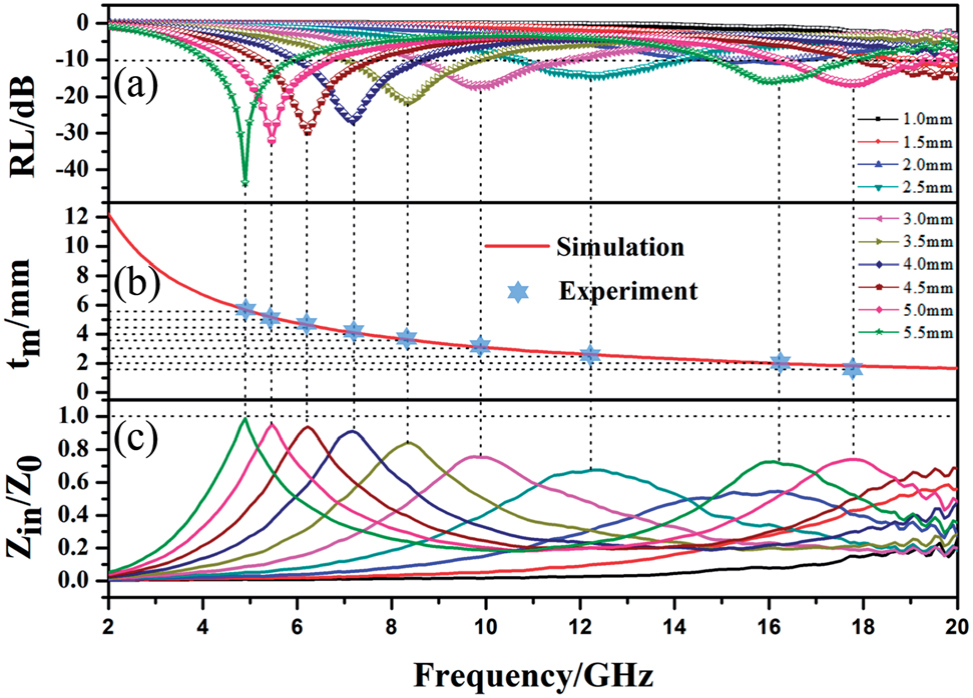
(a) RL–F curves, (b) relationship between simulated thickness and peak frequency, and (c) relationship between *Z*_in_/*Z*_0_ and EMW frequency of MFACs.

To further investigate the EMW absorption property, the relationship between the RL curves, simulated thickness and *Z*_in_/*Z*_0_ and EMW frequency of MFACs was studied. The results are shown in Fig. 8. In the investigated region, the composite exhibited strong EMW loss capacity (RL ≤ −10 dB). When its thickness changed from 1.5–5.5 mm, the minimum RL of the absorbent on the EMW in the frequency range of 4.1–19.2 GHz exceeded −10 dB. At the thickness of 5.5 mm, the minimum RL value achieved was −44.67 dB at 4.9 GHz. Furthermore, the bandwidth corresponding to the RL below −10 dB was 1.7 GHz (from 4.1 to 5.8 GHz). With an increase in the sample thickness, the minimum EMW absorption positions shifted from high frequency to low frequency. This can be explained by the law of the quarter-wavelength matching model.^48^ The relationship between matching frequency (*f*_m_) and matching thickness (*t*_m_) can be calculated using eqn (7), as follows:7
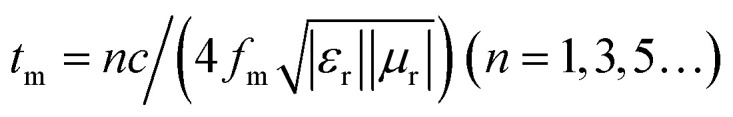
where *c* is the velocity of light. Obviously, *f*_m_ was inversely proportional to *t*_m_. Moreover, the intersection of the perpendicular of the frequency corresponded to the RL peak and the perpendicular of the absorption sample thickness accurately fell on the 11/4 *λ* curve (Fig. 8b), indicating the cancellation of EMW. When the thickness of the material meets the above-mentioned equation at a fixed frequency, the reflection of EMW occurring at the interface between the air and absorbing material is exactly *n*/2 (in which *n* is odd) wave potentials away from the reflection of EMW occurring at the interface between the absorbing material and metal substrate. The two parts of the EMW would be offset, which reduces the reflection of EMW in the material surface.

The low-frequency absorption performance of MFACs was derived from the good impedance matching and strong attenuation due to the synergistic effect of their components and structure.^37^ Good impedance matching can be judged by the extent of the impedance characteristic parameter (*Z*) being close to 1. *Z* was calculated according to eqn (8) and the results are shown in Fig. 8c.8
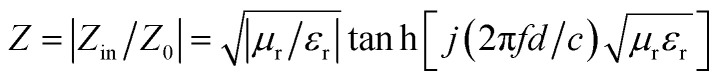


It can be detected that the closer *Z* is to 1, the stronger the RL feature. At the thickness of 4 mm, 4.5 mm, 5 mm, and 5.5 mm, *Z* was close to 1, demonstrating the good impedance matching of the composite material in the frequency range of 4.9–7.2 GHz. This is related to its adequate conductivity and the special structure. Therefore, more EMW in this range can enter the interior of the material to be reduced by EM loss rather than reflected at the material–air interface. This is critical for widening the EAB and enhancing the EMW absorption performance. However, in range of 7.2–17.8 GHz, the value of *Z* slightly deviated from 1, indicating poor impedance matching, which represents the greater reflection of the EMW at the material–air interface in this range. According to the analysis, it was easy to determine that the excellent EMW absorption property of the MFAC in the range of 4.9–7.2 GHz is associated with the good impedance matching, which corresponds to the superior reflection loss values shown in Fig. 7g and 8a.

To further detect the attenuation degree of EMW for the samples, the attenuation factor *α* was calculated using eqn (9)^25^ and the results are shown in Fig. 9.9
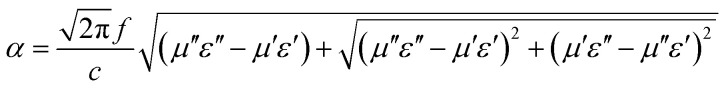


**Fig. 9:**
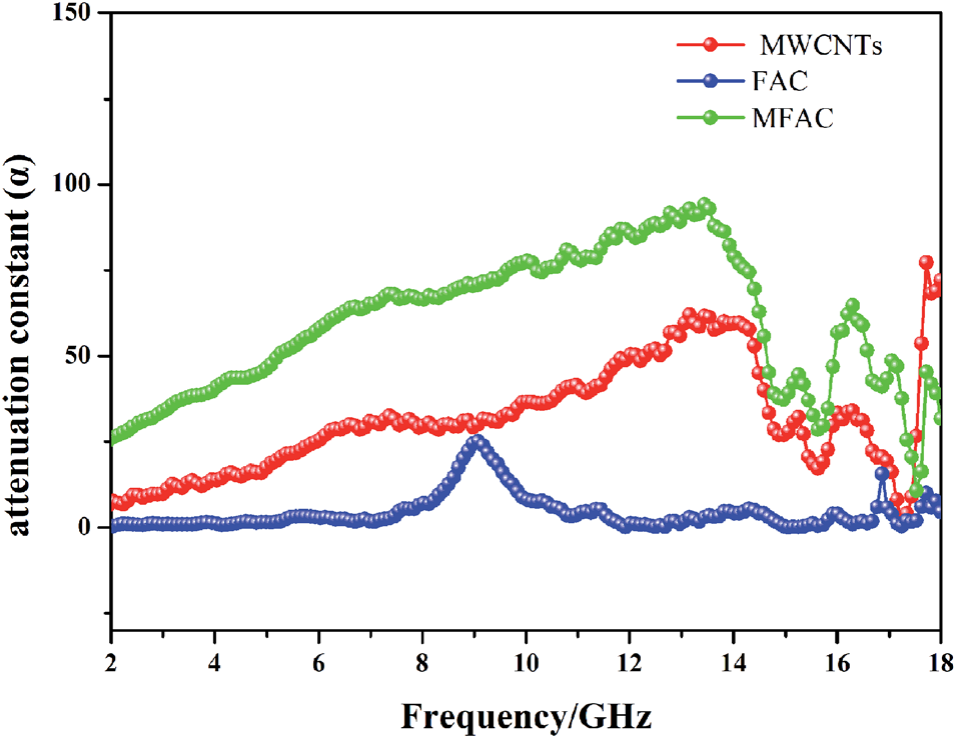
Attenuation constant of all the samples.

It is obvious that the order of the *α* value from big to small was MFACs, MWCNTs and FACs, successively, implying that the new multiple interfacial connected structure contributed more to attenuate EMW. Hence, the favorable impedance matching together with large attenuation ability endowed the composite a high absorption performance.

### The EMW absorption mechanism

**Fig. 10:**
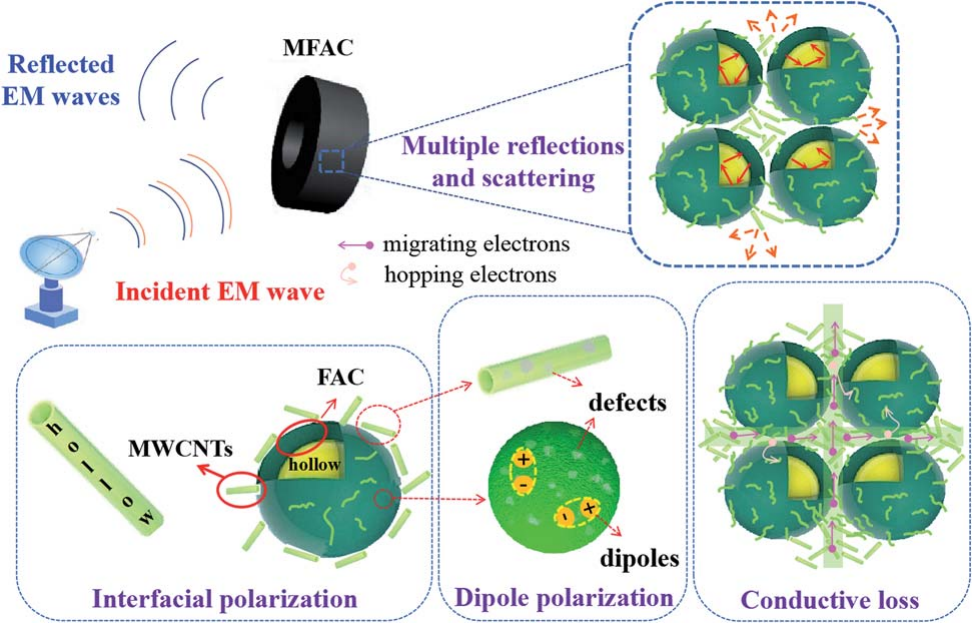
Schematic diagram of the EMW absorption mechanism of the MWCNT/FAC composites.

Therefore, according to the above discussion, the excellent EMW absorption property is illustrated in Fig. 10. The conductive MWCNTs were attached to the surface of FACs with a thin thickness in the portion of gaps formed by the stacking of the insulating spheres. Then, a 3D porous structure of a conductive network was established. This was beneficial for the inner conductive loss. However, due to the thin conductive network, the conductivity was not very high. Thus, good impedance matching could be obtained to guarantee that enough EMW entered the interior rather than be reflected on the surface of the material. When the EMW impinged on the surface of MFACs, a big portion of the microwave energy propagated directly to their interior because of the well-matched impedance and porous network architecture.^49^ The 3D conductive network and plurality of internal interface areas facilitated multiple internal reflections. The internal reflections induced by this unique structure contained three parts. The first one occurred at the single outer shell of the MWCNTs between the different nanotubes. The second was the reflections of the microwaves that reached the inner shell of the MWCNTs. The third presented at the interfaces of MWCNTs and FACs. Some of the microwaves went through the MWCNT layer and permeated the interfaces of MWCNTs and FACs. Due to the different electrical characteristics of these two components, multiple internal reflections occurred. Furthermore, due to the small size of the nanotubes, multiple scatterings occurred. Consequently, effective multiple reflections and scattering occurred during this process. The EMW transmission route was extended, which increased the contact probability of EMW with the absorbing materials, and then facilitated the further dissipation of EM energy. Also, the hollow porous structure endowed the composite material with abundant interfaces. These interfaces greatly enhanced the polarization loss. In addition, the polar functional groups in FACs and the defects in MWCNTs can be regarded as the polarization centre of the dipole to generate dipole polarization and then convert the electromagnetic energy into thermal energy. Finally, the EM reflection was further reduced by the offset between the EMW reflected at the air-absorbing material interface and the EMW reflected at the absorbing material–metal substrate. Therefore, under the synergistic effect of various loss mechanisms, MFACs exhibited an excellent absorption performance.

The EMW absorption ability of typical C-based composites in the recent literature is shown in Table S1.† The optimized MWCNT/FAC composite in this work comprehensively outperformed some of the typical C-based materials in terms of EMW absorption. Thus, these results prove that multi-walled carbon nanotube/fly ash cenosphere composites with the tubular/spherical structural model can be used as effective EMW absorption materials.

## Conclusions

An OH-functionalized multi-walled carbon nanotube/fly ash cenosphere composite with a tubular/spherical structural model was prepared *via* simple physical mixing. The introduction of hollow fly ash cenospheres and the construction of hollow porous structure with multiple interfaces could effectively achieve good impedance matching. The unique porous connected network, multiple interfacial structures, high specific surface area, localized defects and functional groups mainly contributed to the conductive loss, polarization loss and dipole loss of the material to EMW. Owing to the synergistic effect of its components, the OH-functionalized MWCNT/FAC composite exhibited an excellent EMW absorption performance with a minimum RL value of −44.67 dB at 4.9 GHz under an absorbent thickness of 5.5 mm. The effective bandwidth with RL < −10 dB reached 3.62 GHz at a thickness of 2.5 mm. The OH-functionalized MWCNT/FAC hybrid with a complex structure exhibited superior absorption intensity and broader qualified bandwidth than the other typical C-based composites. It is interesting that the simple physical mixing process can achieve such an improved EMW absorption property. Furthermore, the employed raw materials made good use of waste materials and were inexpensive. Thus, this product meets the requirement of high efficiency and simple preparation simultaneously, which greatly enhances its applicability. It also provides practical suggestions to design desirable EMW absorption materials for potential application.

## Author contributions

Mengzhu Liu: conceptualization, methodology, writing – original draft preparation; Hongwei Wang and Yangyang Lv: validation, investigation, visualization; Yingyuan Zhang: data curation, formal analysis; Yongpeng Wang: writing – review & editing, funding acquisition; Haibo Zhang and Zhenhua Jiang: writing – review & editing.

## Conflicts of interest

There are no conflicts to declare.

## Supplementary Material

RA-012-D2RA01960D-s001
