# Supplementary material for: Construction of OH-functionalized MWCNT/solid waste composites with tubular/spherical heterostructures for enhanced electromagnetic wave absorption property
Source: RSC Adv. 2022 May 27;12(25):16003–13. doi: 10.1039/d2ra01960d (PMC9137280; doi:10.1039/d2ra01960d)
Supplement: RA-012-D2RA01960D-s001 [file RA-012-D2RA01960D-s001.pdf]

Electronic Supplementary Material (ESI) for RSC Advances.
This journal is © The Royal Society of Chemistry 2022

# Supplementary Information

# Construction of OH-functionalized MWCNTs/solid waste composites with tubular/spherical heterostructures for the enhanced electromagnetic wave absorption property

Mengzhu Liu,[ab] Hongwei Wang,[a] Yangyang Lv,[a] Yingyuan Zhang,[a] Yongpeng Wang,[*a] Haibo Zhang [b] and Zhenhua Jiang [b]

[a]College of Materials Science and Engineering, Jilin Institute of Chemical Technology, Jilin, 132022, China

[b]College of Chemistry, Jilin University, Changchun, 130012, China

E-mail: wyp4889@163.com

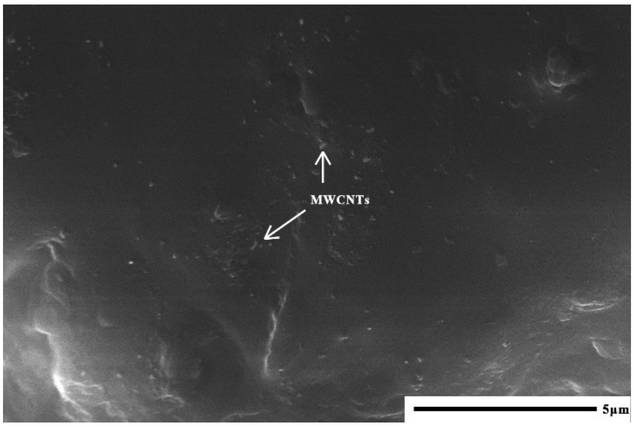


Fig.S1. SEM image of MFACs, in which FAC was broken and the MWCNTs located inside the cenospheres.

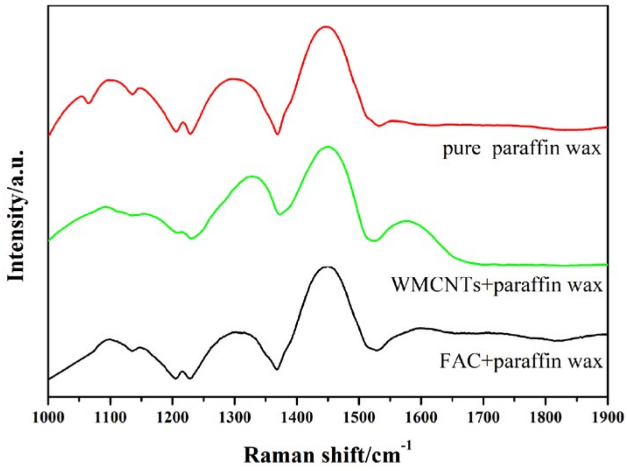


Fig.S2. Raman spectra of different samples.

Table S1. Typical C-based composites for EM wave absorption.

| Sample | $RL_{min}$ (dB) | Optimum Frequency (GHz) | Frequency range (RL≤-10dB) | Refs. |
|---|---|---|---|---|
| CNT/Fe nanocapsulates | -25 | 11 | -- | 50 |
| Porous Co/C Nanocomposites | -35.3 | 5.8 | 5.80 (8.40 −14.20) | 51 |
| Co/CNTs | -36.5 | 4.1 | 1 (3.6-4.6) | 52 |
| porous carbon/Co nanocomposites | -40 | 4.2 | 0.7 (about 3.8-4.5) | 53 |
| $CoFe_2O_4$ hollow sphere/graphene composites | -18.5 | 12.9 | 3.7 (11.3-15) | 54 |
| $Fe_3O_4$/C core-shell nanospindles | -38.8 | 11 | 2.2 (about 9.8-12) | 55 |
| porous RGO/γ-$Fe_2O_3$ composites | -39 | 14.78 | 3.5 (12.5-16) | 56 |
| nano-$Fe_3O_4$ compact-coated CNTs | -43.0 | 15.4 | 8.3 | 57 |
| RGO/ZnO hollow spheres | -45.05 | 9.7 | 3.3 (8.8-12.1) | 58 |
| MWCNTs/FAC composites | -44.67 | 4.9 | 3.62 (10.68-14.30 ) | This work |

**Reference**


50. R. C. Che, L. M. Peng, X. F. Duan, Q. Chen, and X. L. Liang, *Adv. Mater.*, 2004, **16**, 401.
51. Y. Lv, Y. Wang, H. Li, Y. Lin, Z. Jiang, Z. Xie, Q. Kuang, and L. Zheng, *ACS Appl. Mater. Interfaces*, 2015, **7**, 13604.
52. N. Wu, H. Lv, J. Liu, Y. Liu, S. Wang, and W. Liu, *Phys. Chem. Chem. Phys.*, 2016, **18**, 31542.
53. Q. Liu, D. Zhang, and T. Fan, *Appl. Phys. Lett.*, 2008, **93**, 013110.
54. M. Fu, Q. Z. Jiao, Y. Zhao and H. S. Li, J. Mater Chem A., 2014, 2, 735.
55. X. Liu, X. Cui, Y. Chen, X. Zhang, R. Yu, G. Wang, and H. Ma, *Carbon*, 2015, **95**, 870.
56. T. Wang, Y. Li, L. Wang, C. Liu, S. Geng, X. Jia, F. Yang, L. Zhang, L. Liu, B. You, X. Ren, and H. Yang, *RSC Adv*., 2015, **5**, 60114.
57. N. Li, G. Huang, Y. Li, H. Xiao, Q. Feng, N. Hu, and S. Fu, *ACS Appl. Mater. Interfaces*, 2017, **9**, 2973.
58. M. Han, X.Yin, L. Kong, M. Li, W. Duan, L. Zhang, and L. Cheng, *J. Mater. Chem. A.*, 2014, **2**, 16403.
